# Effects of Concomitant Use of Various Psychotropic Medications on the Treatment Response to Transcranial Magnetic Stimulation for Depression: A Literature Review

**DOI:** 10.7759/cureus.72993

**Published:** 2024-11-04

**Authors:** Jordan Intrator, Jack Noto, Renzmark D Vallesteros, Morgan Peltier, John O'Reardon, Muhammad Abbas

**Affiliations:** 1 Psychiatry, NYU Langone Health, Brooklyn, USA; 2 Psychiatry, Hackensack Meridian School of Medicine, Nutley, USA; 3 Psychiatry, Jersey Shore University Medical Center/Hackensack Meridian Health, Neptune, USA; 4 Psychiatry, Psychiatry at the Pavilions, Voorhees, USA

**Keywords:** antipsychotics, benzodiazepines, mood stabilizers, psychostimulants, psychotropic medication, transcranial magnetic stimulation therapy, treatment resistant depression

## Abstract

Many patients are taking psychotropic medications concomitantly with repetitive transcranial magnetic stimulation (rTMS), the latter of which is indicated for the treatment of moderate and severe depressive episodes that have not responded to first-line pharmacotherapy. While preclinical evidence suggests that psychotropic drugs can generally affect rTMS, the specific effect on the clinical response of rTMS for depression is not fully clear. A systematic search of all papers published prior to January 2023 in PubMed, APA, PsycInfo, and Scopus was conducted to identify clinical studies that examine the effects of different psychotropic medications on clinical outcomes in patients undergoing rTMS for depression. A total of 10 articles were identified and extracted for inclusion. This review outlines the results from 10 clinical studies and summarizes the current state of the literature describing rTMS outcomes with concomitant use of several groups of psychotropic agents, including antipsychotics, mood stabilizers, psychostimulants, and benzodiazepines. Antidepressants were excluded from this review due to the evidence base that already exists describing their efficacy with and without rTMS.

## Introduction and background

Introduction

Major depressive disorder (MDD) is a highly prevalent and disabling condition [1]. Even in those who are treated for MDD, a significant portion of patients will show no response to treatment [2-4]. In patients with depression unresponsive to pharmacotherapy, transcranial magnetic stimulation (TMS) has been found to be an effective form of therapy [5, 6].

Although shown to be effective as monotherapy, TMS is often administered to patients simultaneously receiving pharmacotherapy [7]. Studies demonstrate that rTMS is effective and safe in patients taking psychotropic medication [8, 9]. In particular, analyses of studies comparing TMS as monotherapy to TMS as augmentation alongside antidepressant use suggest that TMS is equally effective in each approach for depression [10]. However, the effect that other psychotropic medications including antipsychotics, mood stabilizers, psychostimulants, and benzodiazepines may have on the efficacy of TMS treatment is not fully clear [1] [11].

Psychotropic medications affect cortical excitability and neuroplasticity in a variety of ways [7, 12-16]. While not proven, evidence suggests that TMS mediates its therapeutic effects via induction of neuroplasticity [17, 18]. Evidence shows that different psychotropic agents can significantly alter the neuroplastic effects of TMS, which suggests the potential for deleterious and synergistic effects on treatment responses to TMS when they are administered concomitantly [19-21]. Therefore, it is of clinical importance to determine if certain psychotropic agents significantly affect the treatment response to TMS.

Over the last decade, an increasing number of studies have been published examining the effects different psychotropic medications have on altering the efficacy of TMS therapy. This review aims to summarize this literature, highlighting the existing evidence of the interaction different classes of psychotropic medications may have when administered concurrently with TMS. By doing so we hope to draw attention to this evolving area of study and serve as a foundation for future research that may guide clinical decision-making in patients being treated with TMS.

Methods

A systematic search of PubMed, APA, PsycInfo, and Scopus was conducted to identify studies that examined the effect of psychotropic medications on treatment response to TMS in patients with depressive disorders. The search included all papers published prior to February 2023 and titles and abstracts that included the following search terms: (i) (depression OR major depressive disorder OR MDD) and (ii) (transcranial magnetic stimulation OR TMS) and (iii) (antidepressants OR benzodiazepines OR stimulants OR antipsychotics OR mood stabilizers OR antiepileptics). This search produced a total of 10,049 articles. Inclusion criteria limited inclusion to case-control, cohort, or clinical trial studies involving human subjects, with a study design component that includes concomitant medication use as an independent variable in a population of patients receiving rTMS and with a diagnosis of depression. The exclusion criteria excluded systematic reviews, meta-analyses, case series, case studies, commentaries, editorials, and all non-English publications due to the lack of an interpreter service. This criterion yielded seven articles, following the removal of duplicates. An additional three articles were identified and added following a hand review of the references from the seven articles originally selected. This led to a total of 10 articles included in this review. Several articles that met inclusion criteria examined the effect of multiple psychotropic medications when administered concurrently with TMS. This produced a total of 16 separate outcomes examined.

In order to contextualize and compare the findings of the included studies, a number of demographic, methodological, and outcome characteristics were extracted from each study. These variables included medication class, study type, population size, sex, medications included, primary diagnosis and severity of depressive episode, outcome measure, and results.

Psychotropic drugs were defined as drugs used to treat psychiatric conditions and were classified into the following drug classes: “antipsychotics”, “mood stabilizers”, “psychostimulants”, and “benzodiazepines”. Antidepressants were excluded from this review due to the already existing evidence that describes their efficacy with and without rTMS. We provide a brief summary of this evidence.

## Review

Results and discussion

Five studies were identified that examined the effects of concomitant antipsychotic use and rTMS treatment markers [22-26]. Four studies examined this relationship in a patient concurrently taking mood stabilizers [22, 24, 26, 27]. Two studies investigated whether an association existed in patients taking psychostimulants [24, 28]. Five studies looked at whether an association existed in patients concomitantly taking benzodiazepines [22, 24, 29-31]. The results from each study outcome are outlined and summarized in Table 1, categorized by medication class.

**Table 1:** Demographic variables of included studies *Denotes variables for the entire cohort of the study in cases where medication class-specific variables were not reported, e.g. 64.8% of all the patients included in Bouaziz et al.’s study across all medication classes examined were female. Abbreviations: Li, lithium; VPA, valproic acid; LTG: lamotrigine

Study	Study type	Population size	Age; mean±SD	Sex, % female	Medications included; no. of patients taking each type of medication
Antipsychotics					
Bouaziz et al. (2023) [22]	Retrospective multicenter	171	51.3*±14.9	64.8*	Atypicals
Hebel et al. (2020) [23]	Retrospective multicenter	182	48.9±12.5	50.5	Atypicals: 173; typicals: 9
Hunter et al. (2019) [24]	Retrospective single center	57	46.6*±16.6	54.1*	Atypicals: 56; typicals: 1
Schulze et al. (2017) [25]	Retrospective single center	29	42.0±12.4	75.9	Atypicals: 27; typicals: 2
Fitzgerald et al. (2016) [26]	Retrospective pooled analysis of 11 randomized clinical trials	482	Responders: 47.7*±13.0; Nonresponders: 45.1*±14.0	62.8*	Not described
Mood Stabilizers					
Bouaziz et al. (2023) [22]	Retrospective multicenter	183	51.3*±14.9	64.8*	Li: 97; anticonvulsants: 86
Hebel et al. (2021) [27]	Retrospective single center	299	Li: 47.8; LTG: 53.2; VPA: 48.3; Li + LTG: 49.0	Li: 46; LTG: 61; VPA: 64; Li + LTG: 85	Li: 5; LTG: 18; VPA: 11; Li and LTG: 13
Hunter et al. (2019) [24]	Retrospective single center	73	46.6*±16.6	54.1*	Li: 15; anticonvulsants: 58
Fitzgerald et al. (2016) [26]	Retrospective pooled analysis of 11 randomized clinical trials	330	Responders: 47.7*±13.0; Non-responders: 45.1*±14.0	62.8*	Not described
Psychostimulants					
Wilke et al. (2022) [28]	Retrospective single center	37	44.7±15.9	48.7	Amphetamine: 22; methylphenidate: 9; modafinil: 7
Hunter et al. (2019) [24]	Retrospective single center	56	46.6*±16.6	54.1*	Not described
Benzodiazepines					
Bouaziz et al. (2023) [22]	Retrospective multicenter	204	51.3*±14.9	64.8*	Not described
Deppe et al. (2021) [29]	Retrospective single center	73	49.3±13.3	63	All patients grouped as taking benzodiazepines were only taking lorazepam
Fitzgerald et al. (2020) [30]	Retrospective pooled analysis of 2 parallel superiority clinical trials	64	47.4±13.4*	53.5*	Diazepam: 37; alprazolam: 4; clonazepam: 8; other benzodiazepine:15
Hunter et al. (2019) [24]	Retrospective single center	72	46.6*±16.6	54.1*	Not described
Kaster et al. (2019) [31]	Secondary retrospective analysis of randomized non-inferiority multicenter trial	123	42.3*±11.5	59*	Not described

Antidepressants were excluded from this review due to an already established base of literature. TMS added as an augmentation therapy to antidepressants in patients with depression improves treatment response rates and efficacy of treatment when compared to antidepressant therapy alone [32]. There is no significant change in treatment efficacy when TMS is administered alone or administered concomitantly with antidepressants [10].

Demographic variables at a glance

Most of the projects included were retrospective cohort studies, while the remaining three were secondary retrospective analyses of clinical trials (Table 1). There was moderate variation in population size among outcomes being examined, both within medication class and across medication classes. Age and sex were largely consistent across cohorts with participants on average being middle-aged and leaning female. There was moderate variation in the reporting of medication sub-categories, with the authors of six of the cohorts included failing to report this variable. The impact that variation in demographic variables may have had on the comparability and results of cohorts within each medication class has been discussed further within this article. Methodological and outcome characteristics are detailed in Table 2, categorized by medication class.

**Table 2:** Methodological characteristics and outcomes of included studies MDD: major depressive disorder; MADRS: Montgomery–Åsberg Depression Rating Scale; HDRS17: Hamilton Depression Rating Scale 17 item; HDRS21: Hamilton Depression Rating Scale 21 item; BDI-II: Beck Depression Inventory-II; IDS-SR30: Inventory of Depressive Symptomatology-Self Rated 30 item; QIDS-SR: Quick Inventory of Depressive Symptomatology–Self-Rated; IDS-CR: Inventory of Depressive Symptomatology–Clinician rated; Li: lithium; VPA: valproic acid; LTG: lamotrigine; rTMS: regional transcranial magnetic stimulation; iTBS: intermittent theta burst stimulation

Study	Primary diagnosis/Severity of depressive episode	Outcome measure	Results
Antipsychotics			
Bouaziz et al. (2023) [22]	MDD single episode, MDD recurrent, bipolar disorder with current episode depressive	MADRS scale used; ΔMADRS/ Baseline MADRS ≥50% considered responders. Final MADRS ≤10 considered remitters	The authors found no significant difference in the change in MADRS between patients taking antipsychotics and those who didn’t. There was no significant difference in the proportion of responders and remitters among patients taking antipsychotics versus those who weren't
Hebel et al. (2020) [23]	MDD single episode, MDD recurrent, bipolar disorder with current episode depressive. Further specified as mild-moderate–14.3%; severe–78.9%; psychotic–6.9%	HDRS17 and HDRS21. Decrease in final HDRS of ≥50% considered responders. HDRS-17 or HDRS-21 final scores below 11 or 9, respectively, were considered remitters	The authors found that among the group of patients who weren’t taking antipsychotics, there were at least 10% more responders and remitters compared to the group of patients who were taking antipsychotics
Hunter et al. (2019) [24]	MDD; patients with psychotic symptoms were excluded	IDS-SR30 used. Decrease in final IDS-SR30 ≥50% considered responders	The authors found no significant difference in response to TMS among patients taking antipsychotics and those who weren’t
Schulze et al. (2017) [25]	MDD single episode, MDD recurrent, bipolar disorder with current episode depressive. Patients had at least 2 failed trials of antidepressants. Patients exhibiting psychotic symptoms and active substance use were excluded	HDRS17 and Beck BDI-II used. Decrease in final score ≥50% considered responders	The authors found no significant difference in change from baseline on HDRS17 and BDI-II between patients who were taking antipsychotics compared to those who weren’t
Fitzgerald et al. (2016) [26]	MDD single episode, MDD recurrent, bipolar disorder with current episode depressive, schizoaffective disorder	HDRS 17 was used in 7 studies. MADRS was used in 4 studies. Decrease in final score of ≥50% considered responders	The authors found no significant difference in response to TMS between patients who were taking antipsychotics compared to those who weren’t
Mood Stabilizers			
Bouaziz et al. (2023) [22]	MDD single episode, MDD recurrent, bipolar disorder with current episode depressive	MADRS used ΔMADRS/ baseline MADRS ≥50% considered responders. Final MADRS score ≤10 considered remitters	The authors found that the use of Li was associated with a higher final MADRS score (lower ΔMADRS). This association was not observed with anticonvulsants. Both Li and anticonvulsants had no statistically significant effect on the number of responders or remitters
Hebel et al. (2021) [27]	MDD single episode/MDD recurrent/bipolar disorder with current episode depressive; patients were further specified by severity of depressive episode; mild + moderate/severe/ psychotic. Diagnosis (%): Li, 14/18/68; LTG, 28/6/66; VPA, 40/20/40; Li + LTG: 8/25/67. Severity (%): Li, 10/85/5; LTG, 28/67/5; VPA, 29/42/29; Li + LTG, 18/82/0	HDRS21 used. Decrease of ≥50% considered responders. Final HDRS21 score ≤11 considered remitters.	The authors found that LTG, VPA and Li +LTG demonstrated a superior response to patients who weren’t taking any mood stabilizers. However, they described this association as only at the descriptive level The number needed to treat (NNT) for each anticonvulsant were; LTG (10), VPA (14) and Li + LTG (11) for response and LTG (846), VPA (12) and Li + LTG (5) for remission. The authors noted that Li had an inferior response to no mood stabilizer, with NNT of (−32) for response and (-33) for remission The relative differences in effect sizes between each mood stabilizer regimen and patients not taking mood stabilizers, however, was not significant for each of the mood stabilizers studied.
Hunter et al. (2019) [24]	MDD; patients with psychotic symptoms were excluded.	IDS-SR30 used. Decrease of ≥ 50% considered responders	The authors found that both lithium and anticonvulsants had no significant effect on treatment response to TMS
Fitzgerald et al. (2016) [26]	MDD single episode, MDD recurrent, bipolar disorder with current episode depressive, schizoaffective disorder. Patients were required to have failed at least 2 antidepressant trials.	HDRS 17 was used in 7 studies; MADRS was used in 4 studies. Decrease of ≥50% considered responders.	The authors found that patients receiving anticonvulsants were significantly more likely to respond to rTMS treatment compared to those who weren't taking antiepileptics
Psychostimulants			
Wilke et al. (2022) [28]	MDD; patients with psychotic symptoms were excluded. Patients taking benzodiazepines were excluded.	IDS-SR30 used. The authors measured and analyzed results for each subscale at baseline and conclusion, e.g. "sleep" In addition, they compared and analyzed subscale scores for each distinct psychostimulant medication class.	The authors found that patients who were taking psychostimulants showed significantly improved scores within the IDS-SR30 with regard to “sleep” and “mood/cognition” but not in “arousal/anxiety” when compared to patients not taking psychostimulants. The authors also found that there was a dose-response relationship, with those on lower doses demonstrating the greatest improvement. Amphetamines were found to have a significant effect on "sleep", and also showed a strong trend for “mood/cognition” although this effect was not statistically significant. Methylphenidate was found to have a significant effect on the "mood/cognition" domain, and had no effect on “sleep”. Patients within the Modafinil group were not found to have any significant subscale-specific effects in comparison to patients not taking psychostimulants
Hunter et al. (2019) [24]	MDD; patients with psychotic symptoms were excluded.	IDS-SR30 used. Decrease of ≥50% considered responders.	The authors found that patients who were taking psychostimulants were significantly more likely to respond to TMS compared to nonusers, 39.2% vs. 22.0% respectively
Benzodiazepines			
Bouaziz et al. (2023) [22]	MDD single episode, MDD recurrent, bipolar disorder with current episode depressive.	MADRS used ΔMADRS/ Baseline MADRS ≥50% considered responders. Final MADRS score ≤10 considered remitters.	The authors found that the group of patients who were taking benzodiazepines were less likely to be remitters, 16% vs 25% for patients not taking benzodiazepines. However, the use of benzodiazepines was not found to have a significant effect on ΔMADRS and hence the rate of response.
Deppe et al. (2021) [29]	MDD single episode/MDD recurrent/bipolar disorder with current episode depressive. Patients were also grouped based on the severity of the current depressive episode: mild + moderate/severe/psychotic. Diagnosis: 11/20.5/68.5; severity: 12.5/79.2/8.3. Patients were only included if they were treatment-naive to TMS and absent of any serious somatic illness.	HDRS17 used. Decrease ≥50% considered responders.	The authors found that patients taking lorazepam were significantly less likely to respond to TMS (18%) compared to patients not taking lorazepam (38%). The number of days of lorazepam treatment and dosage were not associated with response to TMS.
Fitzgerald et al. (2020) [30]	MDD with at least one failed medication trial.	MADRS used.	The authors found no significant difference in treatment response, across both rTMS and ITBS, among patients taking benzodiazepines and those who weren’t.
Hunter et al. (2019) [24]	MDD; patients with psychotic symptoms were excluded.	IDS-SR30 used. Decrease of ≥50% considered responders	The authors reported significantly poorer outcomes at 2 weeks for patients receiving benzodiazepines. This result, however, was not significant at 6 weeks. To explore an underlying association further, the study authors performed a secondary analysis including patients taking benzodiazepines as well as patients taking other medications with similar mechanisms of action. This group included other GABA agonists, namely benzodiazepines, nonbenzodiazepines (Z-drugs), baclofen as well as GABAergic anticonvulsants. The patients within this group were found to have significantly poorer responses to rTMS at 6 weeks when compared to patients not taking medications within this class.
Kaster et al. (2019) [31]	MDD; patients were required to have a lack of response to at least one adequate or two inadequate antidepressant trials during the current depressive episode and were TMS naive. Patients were excluded if they were receiving high doses of benzodiazepines, had abused substances within 3 months of study entry, had unstable medical or neurological illness, acute suicidality, diagnosis of bipolar disorder, primary psychotic disorder, psychotic symptoms within the current episode, primary diagnosis of obsessive-compulsive disorder, posttraumatic stress disorder, an anxiety disorder, or a personality disorder; any contraindication to rTMS, previous inadequate trial of ECT, current treatment with anticonvulsants, pregnancy, significant laboratory abnormalities and failure of more than three adequate antidepressant trials.	HDRS17, IDS-CR, and QIDS-SR used. Decrease of ≥50% considered responders. Final HDRS17 <8 considered remitters	Patients were grouped into distinct categories based on response to treatment. "nonresponders" - minimal improvement over treatment; "rapid responders" with near-maximal improvement by Week 2–3, followed by a relative plateau to Week 6; “higher baseline symptoms, linear response”, with steady linear improvement and no apparent plateau by Week 6; and “lower baseline symptoms, linear response” with steady linear improvement and no apparent plateau by Week 6. The authors found that patients taking benzodiazepines were approximately 60% less likely of being rapid responders. Benzodiazepine usage was also associated with a greater likelihood of being within the nonresponse group however this association was not statistically significant. A dose-response trend was observed with higher benzodiazepine usage demonstrating an association with being a nonresponder and lower dosage being associated with being a rapid responder, however, this association was not statistically significant.

Antipsychotics

Five studies examined the association between concomitant use of antipsychotics and treatment response to rTMS therapy for depression. In four out of the five studies, patients were reported as taking either atypical or typical antipsychotics [22-25], Table 1. Among the studies which reported this variable, the overwhelming majority of patients were taking atypical antipsychotics. None of the five studies measured or reported variation in treatment outcomes between atypical or typical antipsychotics or between antipsychotics with distinct mechanisms. 

Four of the studies did not find an association between concomitant antipsychotic use and rTMS treatment outcomes [22, 24-26]. Hebel et al. found that patients taking antipsychotics showed less improvement in depressive symptoms following an rTMS protocol and demonstrated reduced rates of response and remission compared to patients who were not taking antipsychotics while undergoing the protocol [23]. This discrepancy is unlikely to have been due to variation in how response to rTMS was measured or defined between studies, as both Schulze et al. [25], and Fitzgerald et al. [26] used the same scale and definition of response as Hebel et al. [23]. One possible explanation may be the heterogeneity in patient selection between the included studies. Within Hebel et al’s. cohort, for instance, a significant portion of patients were sub-categorized as suffering from a severe depressive episode, 78.9%, or had psychotic symptoms, 6.9% [23]. This is in contrast to other studies within this medication class which explicitly excluded patients exhibiting psychotic symptoms [24-25].

Mood stabilizers 

There were four studies identified in the literature that examined the association between medications that are traditionally classified as mood stabilizers for their role in the treatment of bipolar disorder, spectrum illnesses, and treatment response to rTMS for depression. Three of the included studies further distinguished between patients taking lithium and those taking anticonvulsants [22, 24, 27]; of these, only one study detailed and compared the specific anticonvulsants patients were taking [27].

Hebel et al. reported that patients taking anticonvulsive agents while undergoing rTMS treatment for depression demonstrated higher response and remission rates [27]. However, these differences were not clinically significant for each of the anticonvulsants for both response to rTMS treatment and remission of depressive symptoms, Table 2. Hunter et al. found no relationship between concomitant use of anticonvulsants and rTMS treatment outcomes in an observational study of patients with MDD who underwent a six-week course of rTMS [24]. A multicenter retrospective analysis in patients with treatment-resistant depression who received rTMS therapy yielded similar findings, with no association detected between concomitant use of anticonvulsants and treatment response rates, remission rates, or overall symptom reduction [22]. However, the specific anticonvulsive agents that were examined were not detailed in this study.

Hebel et al. found that lithium was associated with a negative number needed to treat when compared to no mood stabilizer for both response to rTMS and remission of depressive symptoms, Table 2. However, this association was not statistically significant, with study authors calculating that a sample size of 5,566 would be needed in order to reach significance [27]. Bouaziz et al. found that concomitant lithium use was associated with a reduction in symptom improvement, Table 2. However, the study authors also showed that lithium did not appear to affect overall response or remission rates [22].

Finally, a pooled analysis of data collected from 11 clinical trials found that patients concomitantly taking any mood-stabilizing agent while undergoing rTMS for depression demonstrated increased rates of treatment response [26]. The underlying reason for this discrepancy was not clear, with several studies which did not find such an association [22, 27], utilizing similar outcome measures and definitions of treatment response (Table 2). One possible explanation may be that the patient population included in Fitzgerald et al. could have been more treatment-resistant than the cohorts of other studies, with patients required to have failed at least two antidepressant trials [26]; additionally, the authors noted that the majority of their cohort had failed multiple (more than two) medication trials.

Psychostimulants

Two retrospective studies examined the effects of psychostimulants on rTMS outcomes in patients with depression [24, 28]. Willke et al. further detailed the type of psychostimulant patients were taking and the outcomes for each medication [28]. Both studies found that concomitant use of psychostimulants was associated with more robust rTMS treatment outcomes [24, 28]. Hunter et al, found that the proportion of responders among patients taking psychostimulants was approximately 17% greater than that of nonusers, Table 2 [24]. Willke et al. reported subscale improvements at the end of treatment in “mood/cognition” and “sleep” but not in “anxiety/arousal” for patients taking psychostimulants when compared to those who weren’t, Table 2. The authors also explored a dose-response relationship in patients taking amphetamines [28], finding that patients on lower doses of amphetamines demonstrated improved outcomes compared to those on higher doses. They also found that patients taking amphetamines had significantly improved outcomes with regard to the “sleep” domain compared to patients not taking psychostimulants. Methylphenidate, in contrast, was associated with significantly improved “mood/cognition” after rTMS treatment. Patients taking modafinil were not found to differ significantly in any of the IDS-SR30 domains at the end of treatment compared to nonusers. The authors suggested an underlying mechanism of action for the results observed may be a synergistic effect between rTMS and psychostimulants whereby, low dose psychostimulants may enhance norepinephrine and dopamine release, augmenting the effects of rTMS and increasing neural plasticity. Willke et al. suggested that based on these findings, low-dose psychostimulant administration may have a role in enhancing patient response to TMS [28].

Benzodiazepines

Five articles examined the association between concomitant benzodiazepine use and clinical outcomes of rTMS treatment for depression. One study detailed the specific benzodiazepines patients were taking [30]. While all patients taking benzodiazepines included in the Deppe et al. cohort were taking lorazepam [29]. Four of the five articles describe studies that suggest a negative association between concomitant benzodiazepine use and clinical response to rTMS [22, 24, 29, 31]. Three of these four articles describe a direct association between benzodiazepine use and poorer markers of treatment outcome, including reduced response rates, reduced remission rates, and reduced improvement in symptomatology [22, 24, 29]. The other study demonstrated an indirect association between benzodiazepines and attenuated response to rTMS, finding that patients who were not taking benzodiazepines while undergoing rTMS were significantly more likely to demonstrate a “rapid response” to treatment [31]. However, an analysis of pooled data from two clinical trials described by Fitzgerald et al. found no difference in the change in MADRS scores or in response rates between patients taking benzodiazepines and patients not taking benzodiazepines while undergoing an rTMS protocol for depression [30]. This discrepancy was not explained by the methodological or demographic variables extracted within the present study. Bouaziz et al., for instance, utilized the same outcome measure in Table 1, [22, 30]. Kaster et al. provided the same length of rTMS treatment [30, 31].

Limitations 

All 10 studies included in this review were retrospective analyses, thus significantly limiting the degree of control over potential confounders. Currently, there are no prospective randomized controlled trials that address the effect of different classes of concomitant medications on therapeutic outcomes in patients undergoing rTMS for depression.

A major confounding variable present in most of the studies reviewed is the possibility that the subjects who entered the study on specific psychotropic agents had fundamentally different depressive phenotypes than the subjects who did not enter the study on these agents. For example, the enhanced response to rTMS seen in patients on psychostimulants can be attributed to the fact that patients with depression who are treated with psychostimulants have a depressive phenotype that is more likely to respond to rTMS than other depressive phenotypes.

Other limitations include the heterogeneity observed across the studies. For example, there was a range of different rTMS protocols employed in each study. Therefore, it is unclear whether the findings were specific to the rTMS protocol used in each specific study or whether the findings can be generalizable across a range of different protocols. Additionally, the spectrum of depressive disorders included in the samples also varied significantly. Some studies used samples of patients with MDD, while other studies’ samples included patients with both unipolar depression and bipolar depression.

While not a methodological limitation, it is worthy to note that most of the effect sizes of these studies were small. This could limit the clinical relevance of some of these studies’ findings. However, it is still not possible to determine a true effect size until more controlled studies are conducted.

## Conclusions

In conclusion, there is significant variation regarding the effect of concomitant psychotropic medication use among patients undergoing TMS for depression. Sorting by medication class, antipsychotic use may attenuate the response to TMS among patients suffering from a severe depressive episode or among patients exhibiting psychotic symptoms. However, there is limited evidence favoring this possibility and more evidence supporting a negligible effect on TMS response. Similarly, at present there is limited evidence suggesting any meaningful effect on response among patients taking mood stabilizers alongside TMS. In contrast, the existing literature consistently points to an association between psychostimulant use and improved treatment response to TMS. These findings, however, are limited by the small population sizes of the existing studies and their use of patient-rated scales. Among the psychotropic medications explored within the present study, benzodiazepines exhibit the strongest evidence for an attenuating effect on treatment response to TMS.

It is abundantly clear from this conglomerate of retrospective studies that controlled, prospective clinical trials are needed and called for to better understand the effect and clinical significance of different psychotropic agents on treatment markers of rTMS therapy for depression. While retrospective analyses have an integral role in strengthening clinical hypotheses and providing preliminary evidence for an underlying association, they are limited insofar as their ability to outline the true effects. Until these studies are complete, the associations or lack of associations found in these studies between drug type and treatment measures are limited to being just a correlative relationship.
